# Predicting outcomes for locally advanced rectal cancer treated with neoadjuvant chemoradiation with CT-based radiomics

**DOI:** 10.1038/s41598-022-10175-2

**Published:** 2022-04-13

**Authors:** Fuqiang Wang, Boon Fei Tan, Sharon Shuxian Poh, Tian Rui Siow, Faye Lynette Wei Tching Lim, Connie Siew Poh Yip, Michael Lian Chek Wang, Wenlong Nei, Hong Qi Tan

**Affiliations:** Division of Radiation Oncology, National Cancer Centre Singapore, Singapore, Singapore

**Keywords:** Cancer imaging, Gastrointestinal cancer

## Abstract

A feasibility study was performed to determine if CT-based radiomics could play an augmentative role in predicting neoadjuvant rectal score (NAR), locoregional failure free survival (LRFFS), distant metastasis free survival (DMFS), disease free survival (DFS) and overall survival (OS) in locally advanced rectal cancer (LARC). The NAR score, which takes into account the pathological tumour and nodal stage as well as clinical tumour stage, is a validated surrogate endpoint used for early determination of treatment response whereby a low NAR score (< 8) has been correlated with better outcomes and high NAR score (> 16) has been correlated with poorer outcomes. CT images of 191 patients with LARC were used in this study. Primary tumour (GTV) and mesorectum (CTV) were contoured separately and radiomics features were extracted from both segments. Two NAR models (NAR > 16 and NAR < 8) models were constructed using Least Absolute Shrinkage and Selection Operator (LASSO) and the survival models were constructed using regularized Cox regressions. Area under curve (AUC) and time-dependent AUC were used to quantify the performance of the LASSO and Cox regression respectively, using ten folds cross validations. The NAR > 16 and NAR < 8 models have an average AUCs of 0.68 ± 0.13 and 0.59 ± 0.14 respectively. There are statistically significant differences between the clinical and combined model for LRFFS (from 0.68 ± 0.04 to 0.72 ± 0.04), DMFS (from 0.68 ± 0.05 to 0.70 ± 0.05) and OS (from 0.64 ± 0.06 to 0.66 ± 0.06). CTV radiomics features were also found to be more important than GTV features in the NAR prediction model. The most important clinical features are age and CEA for NAR > 16 and NAR < 8 models respectively, while the most significant clinical features are age, surgical margin and NAR score across all the four survival models.

## Introduction

Colorectal cancer is the fourth commonest cancer and the second leading cause of cancer-related death worldwide^1^. In patients with locally advanced rectal cancer (LARC), multidisciplinary treatment involving neoadjuvant chemoradiotherapy (NACRT) followed by total mesorectal excision (TME) has been the standard of care^2^. Neoadjuvant options include short course radiotherapy (SCRT) alone^3^, SCRT with chemotherapy^4–6^ or long course chemoradiation before or after systemic treatment^7–9^, each with its associated risks and benefits. Treatment strategies and responses remain heterogenous and the current selection strategies are not robust. Real world data suggests that we may be overstaging patients and this may lead to the exposure of a more intensified treatment strategy and their associated toxicities^10^. Classically, about 20% of patients achieve pathological complete response (pCR) after NACRT^11^ and with the recent PRODIGE and RAPIDO trials, this rate is higher at 28%^11,12^. With these patients, the non-operative watch-and-wait approach to spare morbidity without sacrificing disease control may have been a reasonable option^13,14^. Hence, the ability to risk stratify patients and predict outcomes to guide treatment strategies pre-operatively would be beneficial.

There are various surrogate markers that predict well for survival outcomes for treatment in LARC. These include magnetic resonance imaging (MRI) post NACRT, neoadjuvant pathologic stage groups (ypTNM), neoadjuvant rectal (NAR) score, tumour regression grade (TRG) and pCR^15–19^. However, these markers rely on postoperative findings to predict outcome and so, are not useful agents to counsel patients preoperatively. Radiomics is an emerging innovation with promising utility as a non-invasive imaging biomarker for tumour response and can be used in LARC pre-operatively to guide treatment strategies. It involves extraction and analysis of radiological image features from conventional imaging to assess for tumour characteristics such as texture, shape and heterogeneity^20^. Recently, more complex prediction models using machine learning classifiers and deep learning have been developed with its oncological application in the prediction of pathology, genomics, therapeutic response and clinical outcomes^21^.

Radiomics prediction models for LARC are mainly MRI-based with heterogenous protocols and imaging parameters, which may limit the applicability and reproducibility of these models^22–27^. Computed tomography (CT) images may be more uniform and easily available. Most radiomics models also mainly predicted for pCR, which is a widely used surrogate for endpoints such as improved local control^25,28,29^. The NAR score on the other hand has a greater predictive ability than pCR for overall survival (OS) and has been proposed by the NRG oncology as a primary end point to assess preoperative treatment efficacy in clinical trials in rectal cancer^19^. Previous CT- and MRI-based radiomics studies in LARC have not correlated the predicted NAR score with clinical outcomes. Thus, in devising a predictive model, both NAR score and long term outcomes should be accounted for to allow future applicability of the radiomics model where there is confidence in both the short and long term outcomes.

The primary aim of our study was to investigate the predictive role of a radiomics model for the neoadjuvant rectal (NAR) score based on the pre-treatment radiotherapy planning contrasted CT of patients with LARC undergoing neoadjuvant treatment. The secondary aim was to investigate the predictive role of a radiomics model on locoregional failure free survival (LRFFS), distant metastasis free survival (DMFS), disease free survival (DFS) and overall survival (OS).

## Materials and methods

### Patients and endpoints

This is a single centre, retrospective study involving patients who had locally advanced rectal adenocarcinoma and received neoadjuvant chemoradiation with subsequent TME between 2006 and 2017. Retrospective chart review of these patients was conducted for basic demographic, disease staging (based on the AJCC, 7th edition) chemoradiation and surgical details as well as the pathology where available. The CT data from 191 patients were used for this radiomics study.

The endpoints of interest were NAR score, locoregional failure free survival (LRFFS), distant metastasis free survival (DMFS), disease free survival (DFS) and overall survival (OS). The NAR score was calculated and two different binary outcomes based on NAR > 16 (high risk) and NAR < 8 (low risk) were used in this work^19^. The extreme range of the NAR was chosen as it represents the best and worst survivorship. This study was approved with waiver of informed consent from Singhealth centralised institution review board and all methods were performed in accordance with relevant guidelines and regulations.

### Image acquisition, segmentation and radiomics feature extraction

Images with contrast were captured with two different CT scanners located in the centre’s radiotherapy department. The first CT scanner was the GE LightSpeed RT16 and the second was Siemens SOMATOM definition AS. All images were acquired with 120 kVp X-ray with slice thickness 2.0 mm (Siemens scanner) and 2.5 mm (GE Scanner). The default standard and B31f convolution kernels were used for the GE and Siemens scanner respectively. The in-slice resolution was 512 by 512 for all images. The patients were allocated randomly to the two different CT scanners subjected to the availability of the scanners.

The segmentations were performed manually by the radiation oncologist (F. Q. Wang) without knowledge of the pathologic outcome of the patient. Two segmentations consisting of the primary tumour (GTV) and mesorectum (CTV) were contoured using the CT image and there were no overlaps in these segmentations. These segmentations were used for shape calculation and was known as the *morphological mask* in Image Biomarker Standardization Initiative (IBSI)^30^. The manually contoured segmentations were subsequently re-segmented to remove any voxel with HU below -50 HU. This was to remove part of contours which encompassed the air in the rectum and was known as the *intensity mask* in IBSI.

The radiomics features were extracted using Pyradiomics v3^31^. It comprised of shape, first order and second order textural features (GLRLM, GLSZM, NGDTM and GLCM). These features were IBSI compliant^30^. The CT image was first interpolated with 1 mm isotropic voxel before feature extraction^32,33^. The radiomics features were extracted from the original CT image and the filtered CT image. LoG (Laplacian of Gaussian) filter with sigma 1.0, 2.0 and 3.0 mm and wavelet filters were applied on the image. This gives a total of 1130 radiomics features per CTV and GTV segmentation. A constant bin width of 10 HU was used for textural calculation. The bin width was chosen to give between 16 to 128 bins for the calculation of textural features. The resulting numbers of bins should be large enough to capture the heterogeneity within the ROI and small enough to be insensitive to the noise within the image^34,35^.

We designed a procedure to select a subset of features from the original 1130 radiomics features which are robust to CT scanner variation and inter-rater variation in CTV and GTV contouring^36^. The contouring variability is simulated by performing morphological dilatation and erosion operations of up to 2.0 mm on the segmentation (this generated four additional structures per patient). The CT scanner variability is simulated by adding an independent Gaussian, Poisson and Uniform noise to the CT image where the mean of the Gaussian, Poisson and Uniform noise distributions and the standard deviation of the Gaussian and Uniform distribution were both 20 HU. These parameters were estimated from a phantom study on the two CT machines in the institution as shown in Figs. S1 and S2. The process of adding the noise to the original CT image was repeated five times for each distribution, resulting in fifteen different CT image for each patient. Overall, the morphological operations and noise addition generated 19 additional sets of radiomics features per patient. Details of the measurement are shown in the Figs. S1 and S2. Intraclass Correlation Coefficients (ICC) with a threshold of 0.7 is used to select the robust features. This yields a final set of 404 and 254 robust radiomics features for the GTV and CTV respectively. The radiomics features from the CTV and GTV were combined to form the radiomics features for model building in the next section. It is interesting to note that this is not the only method to instill robustness in the model. Other method to instill robustness to contouring variability include removing radiomics features that are correlated to the GTV/CTV volumes or having different readers to contour the CT and eventually selecting features which are insensitive to the different readers. The latter method requires access to manpower hours—a luxury not all centers can afford.

### Model building: constructing the NAR and survival model

**Figure 1:**
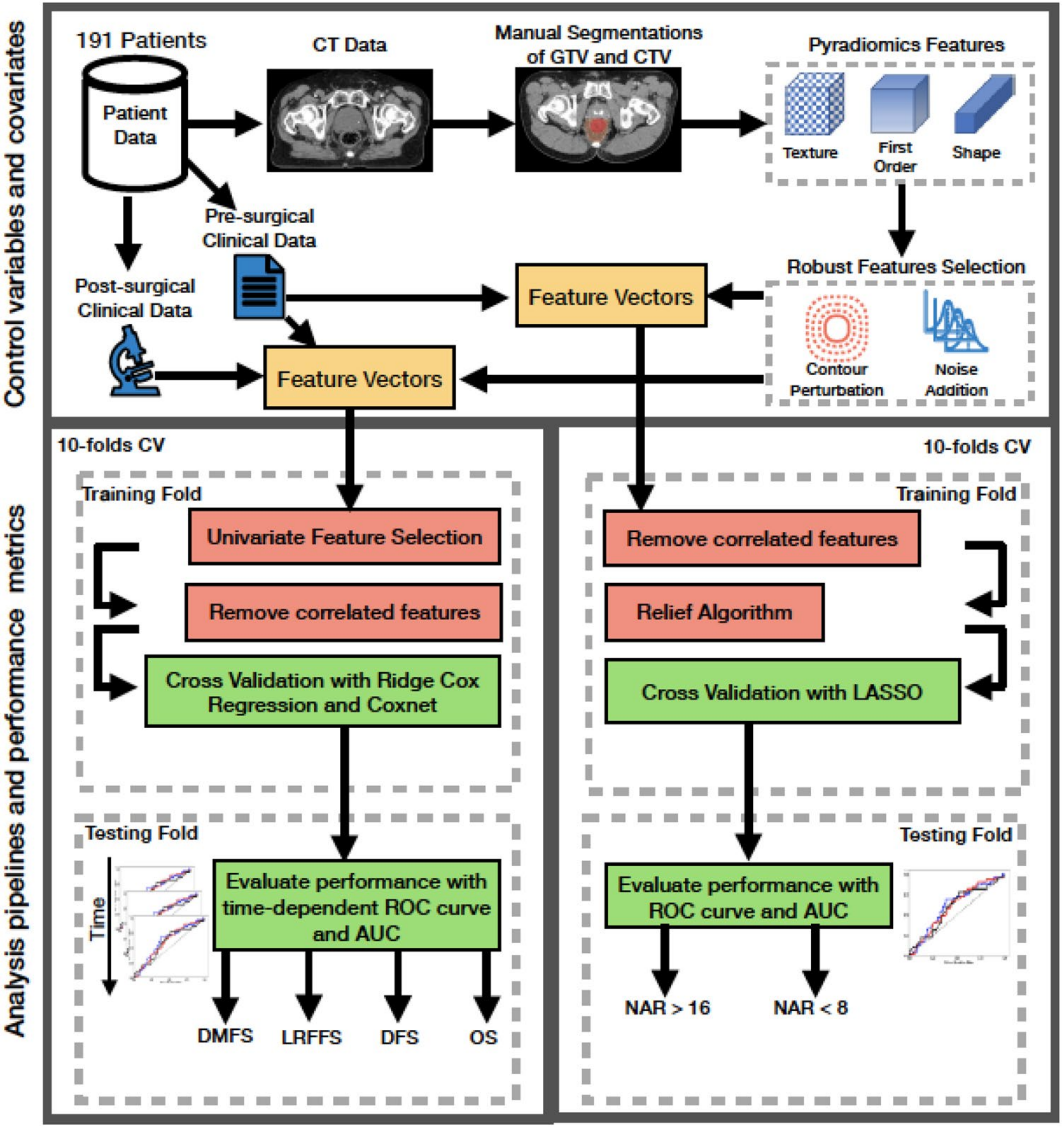
The schematics of the covariates and analysis pipeline used in this manuscript. The covariates are used to construct 4 survival models (DMFS, LRFFS, DFS, OS) and 2 binary classification models (NAR > 16 and NAR < 8). Pre-surgical and radiomics features are used to construct the NAR models while an additional post-surgical features are used for the survival models.

Two prediction models were constructed, namely the NAR model which is based on radiomics and clinical features available pre-operatively and the survival model which is based on radiomics and both clinical and pathologic features available pre- and post-operatively. The analysis pipelines for the NAR and survival model are illustrated in Fig. 1. A nested cross validation approach is used to ensure an objective manner of choosing the parameter λ, and to reduce both variance and bias in the model compared to using a single internal hold-out test set. All statistical tests and analyses were performed using R statistical software (version 3.4.2; R Foundation for Statistical Computing)^37^. A two-sided *P*-value < 0.05 was considered significant.

#### NAR modelling

The NAR model was constructed from radiomics and clinical features available pre-operatively. The clinical features comprised of cT, cN and carcinoembryonic antigen (CEA) values at diagnosis. cT, cN and CEA were regarded as a continuous variable in this model. Feature reduction was performed by firstly, removing correlated features with Spearman correlation greater than 0.60 and secondly, using Relief Algorithm. The final subset of radiomics and clinical features were input into LASSO (Least Absolute Shrinkage and Selection Operator) algorithm to construct the final combined model. The optimal lambda parameter in the LASSO model was selected based on minimum deviance from tenfold cross validation. To compare the contribution of the GTV and CTV features towards NAR prediction, we calculated the global feature importance using DALEX toolbox in R. Each of the feature has its values randomized one at a time, and the loss function value of the model was compared to the original loss function value of the full model. The difference in the value of the loss function is an indication of the importance of the feature in the model. The union of the set of features after the feature selection phases for all the ten folds were fed into the DALEX pipeline for calculating global feature importance. The final NAR models to be used in actual clinical testing is obtained by running the entire pipeline above through the entire dataset. The probability cutoff is selected using the Youden’s index^38^ and the sensitivity, specificity, positive predictive value (PPV) and negative predictive value (NPV) are reported.

#### Survival modelling

The OS, DFS, DMFS and LRFFS survival models were constructed from (1) radiomics features, (2) clinicopathologic features and (3) combination of both radiomics and clinicopathologic features. These result in three different models for each survival type. Robust radiomics features were first selected based on the method outlined in previous section. Univariate analysis with Cox Proportional Hazard Regression was performed on the radiomics, clinical and combined features in each model. Significant features with log-rank test *P* < 0.1 were retained in the model. Due to a large dimensional radiomics feature space, a further feature reduction technique based on retaining uncorrelated features was performed (removing features with Spearman correlation greater than 0.6). The radiomics model was constructed with Cox regression with a L1 regularization term (LASSO). The linear predictor from the radiomics model was defined as the “radiomics score” and was added to the significant clinical features to form the features for the combined model. The “radiomics score” approach is used in survival modelling due to the large number of clinical predictors as compared to in NAR models, and this approach could better lead to a parsimonious model. Both the clinical and combined models were constructed with Cox regression with a L2 regularization term (ridge regression). The optimal lambda parameter in the LASSO and ridge regression model was selected based on minimum deviance from tenfold cross validation. The regularized Cox regression was performed using *glmnet_*2.0–18 package in R^39^. The LASSO and ridge regularization are selected by setting α = 1 and α = 0 respectively.

### Assessing model performance

A nested tenfold cross validation was used to assess the performance of the NAR and survival model. Cross validation was employed in the training fold to determine the optimal parameters for the LASSO and ridge regression models as outlined in the previous section. The fold was selected to have the same number of events within. Feature reductions were applied strictly to the training fold to ensure no data leakage. The performance of the training model for NAR applied to the testing fold was evaluated using AUC. A final single ROC curve and AUC value was obtained for the NAR model by averaging over all ROC curves from the 10 folds. The performance of the training model on the four survival types were evaluated on the testing fold by using time-dependent AUC^40^. A final integrated AUC across all time point was reported for each testing fold. The time-dependent AUC was calculated using *survAUC_1.0–*5 package in R. The AUCs of the clinical model was then compared to the combined model using pairwise *t-test* with Bonferroni’s correction for multiple testing, to show if the combined model performs better than pure clinical model.

## Results

### Patients and endpoints

In total, 191 patients were included in the study. There were 140 men (73.3%) and 51 women (26.7%). The median age was 63 years (range, 28 to 85). There were 11, 141 and 39 patients with T2, T3 and T4 initially staged rectal tumors, respectively. Ninety percent (168/191) of the patients had initial CEA levels available, ranging from 0.8 to 709.8 ng/mL (IQR 2.7–16.025; median, 5.9 ng/mL). The missing CEA levels were imputed using the median value across the available data. The median follow-up time was 60 months. The patient baseline characteristics are summarized in Tables 1 and 2.

**Table 1:** Baseline NAR characteristics of the patients.

	No. of patients	NAR > 16			NAR < 8		
		No (%)	OR (95% CI)	*P*-value	No (%)	OR (95% CI)	*P*-value
Total no. of patients 62.6 (11.7)	191	74	–	–	36	–	–
**Clinical diagnosis**							
Age, years	191	62.6 (11.7)	1.01 (0.98 to 1.04)	0.477	58.8 (11.5)	0.97 (0.93 to 1.00)	0.046*^,^^†^
cT stage							
T2	11	3 (4.05)	Ref.	Ref.	3 (8.33)	Ref.	Ref
T3	141	58 (78.4)	1.80 (0.49 to 8.97)	0.393	29 (80.6)	0.67 (0.18 to 3.40)	0.597
T4	39	13 (17.6)	1.30 (0.30 to 7.09)	0.737	4 (11.1)	0.31 (0.05 to 1.97)	0.203
cN stage							
N0	43	8 (10.8)	Ref.	Ref.	11 (30.6)	Ref.	Ref.
N1	110	47 (63.5)	3.20 (1.41 to 8.08)	0.005*^,^^†^	21 (58.3)	0.69 (0.30 to 1.63)	0.384
N2	38	19 (25.7)	4.25 (1.60 to 12.2)	0.003*^,^^†^	4 (11.1)	0.35 (0.09 to 1.17)	0.091*
CEA at diagnosis	191	21.6 (41.6)	1.00 (1.00 to 1.00)	0.740	4.23 (5.76)	0.92 (0.86 to 0.99)	0.019*^,^^†^
**Treatments**							
Adjuvant chemotherapy							
Yes	144	58 (78.4)	Ref.	Ref.	29 (80.6)	Ref.	Ref.
No	47	16 (21.6)	0.77 (0.38 to 1.52)	0.455	7 (19.4)	0.71 (0.26 to 1.67)	0.441
Surgical margin							
Close	8	2 (2.70)	0.55 (0.07 to 2.58)	0.470	2 (5.56)	1.31 (0.17 to 6.22)	0.763
Positive	26	11 (14.9)	1.16 (0.48 to 2.69)	0.738	1 (2.78)	0.17 (0.01 to 0.86)	0.028*^,^^†^
Clear	157	61 (82.4)	Ref.	Ref.	33 (91.7)	Ref.	Ref.
Time RT to surgery, days	191	69.1 (26.0)	1.00 (0.99 to 1.00)	0.572	73.9 (61.3)	1.00 (0.99 to 1.01)	0.820
**Pathology**							
pT stage							
T0	27	1 (1.35)	Ref.	Ref.	25 (69.4)	Ref.	Ref.
T1	11	0 (0.00)	2.31 (0.06 to 95.3)	0.615	9 (25.0)	0.35 (0.03 to 3.77)	0.361
T2	34	11 (14.9)	10.8 (1.84 to 279)	0.005*^,^^†^	2 (5.56)	0.01 (0.00 to 0.04)	< 0.001*^,^^†^
T3	99	48 (64.9)	20.8 (4.16 to 506)	< 0.001*^,^^†^	0 (0.00)	0.00 (0.00 to 0.01)	< 0.001*^,^^†^
T4	19	14 (18.9)	48.3 (7.53 to 1330)	< 0.001*^,^^†^	0 (0.00)	0.01 (0.00 to 0.05)	< 0.001*^,^^†^
**pN stage**							
N0	116	2 (2.70)	Ref.	Ref.	35 (97.2)	Ref.	Ref.
N1	61	58 (78.4)	853 (177 to 7709)	< 0.001*^,^^†^	1 (2.78)	0.05 (0.00 to 0.22)	< 0.001*^,^^†^
N2	13	13 (18.9)	533 (69.6 to 15,143)	< 0.001*^,^^†^	0 (0.00)	0.19 (0.01 to 1.03)	0.055*

*P < 0.1, ^†^*P* < 0.05.

The data are shown as the number (percentage) or median (interquartile range).

*CEA* carcinoembryonic antigen.

**Table 2:** Baseline survival characteristics of the patients.

	No. of patients	LRFFS		DMFS		DFS		OS	
		5 years	P-value	5 years	P-value	5 years	P-value	5 years	P-value
Total no. of patients	191								
**Clinical diagnosis**									
cT stage			0.031*^,^^†^		0.075		0.051		0.062
T2	11	100		100		100		100	
T3	141	79.7		80.0		79.9		79.6	
T4	39	60.4		67.3		62.4		64.9	
cN stage			0.304		0.272		0.280		0.250
N0	43	87.4		87.8		87.5		86.7	
N1	110	71.4		73.1		71.6		72.7	
N2	38	83.0		81.4		83.7		81.5	
**Treatments**									
Adjuvant chemotherapy			0.035*^,^^†^		0.063*		0.050*		0.048*^,^^†^
Yes	144	80.0		81.6		80.6		80.7	
No	47	67.6		68.4		67.6		68.1	
Surgical margin			< 0.001*^,^^†^		< 0.001*^,^^†^		< 0.001*^,^^†^		< 0.001*^,^^†^
Close	8	70.0		75.0		75.0		72.9	
Positive	26	41.7		46.8		36.5		48.5	
Clear	157	82.5		83.3		83.1		82.5	
**Pathology**									
pT stage			0.001*^,^^†^		< 0.001*^,^^†^		< 0.001*^,^^†^		< 0.001*^,^^†^
T0	27	100		100		100		100	
T1	11	90.0		90.0		90.0		90.0	
T2	34	89.7		90.9		90.9		90.0	
T3	99	68.7		71.4		69.8		70.9	
T4	19	58.1		57.2		55.1		49.7	
pN stage			0.002*^,^^†^		0.001*^,^^†^		0.001*^,^^†^		0.003*^,^^†^
N0	116	85.0		86.7		85.2		86.0	
N1	61	67.1		67.0		66.8		67.7	
N2	13	55.6		55.1		55.1		55.6	

*P < 0.1, ^†^*P* < 0.05.

The 5-years survivals are expressed as percentage. The *P-*values are calculated from the log-rank test of the stratified Kaplan–Meier curves.

In terms of neoadjuvant chemotherapy, all patients received concurrent capecitabine, or 5-flourouracil. All but one patient received 3DCRT to a dose of 50.4 Gy in 28 daily fractions delivered in two phases. The median interval between completing neoadjuvant treatment and surgery was 71 days (range, 38 to 315 days; IQR, 62–91). 77% (147/191) of patients had a low anterior resection, and 23% (44/191) had an abdominoperineal resection. ypT was 0, 1, 2, 3, and 4 in 14% (27/191), 6% (11/191), 18% (34/191), 52% (99/191), and 10% (19/191) of patients, respectively. ypN was 0, 1, and 2 in 61% (116/191), 32% (61/191), and 7% (13/191) of patients, respectively. 14% (27/191) of patients had a pCR after NACRT.

**Figure 2:**
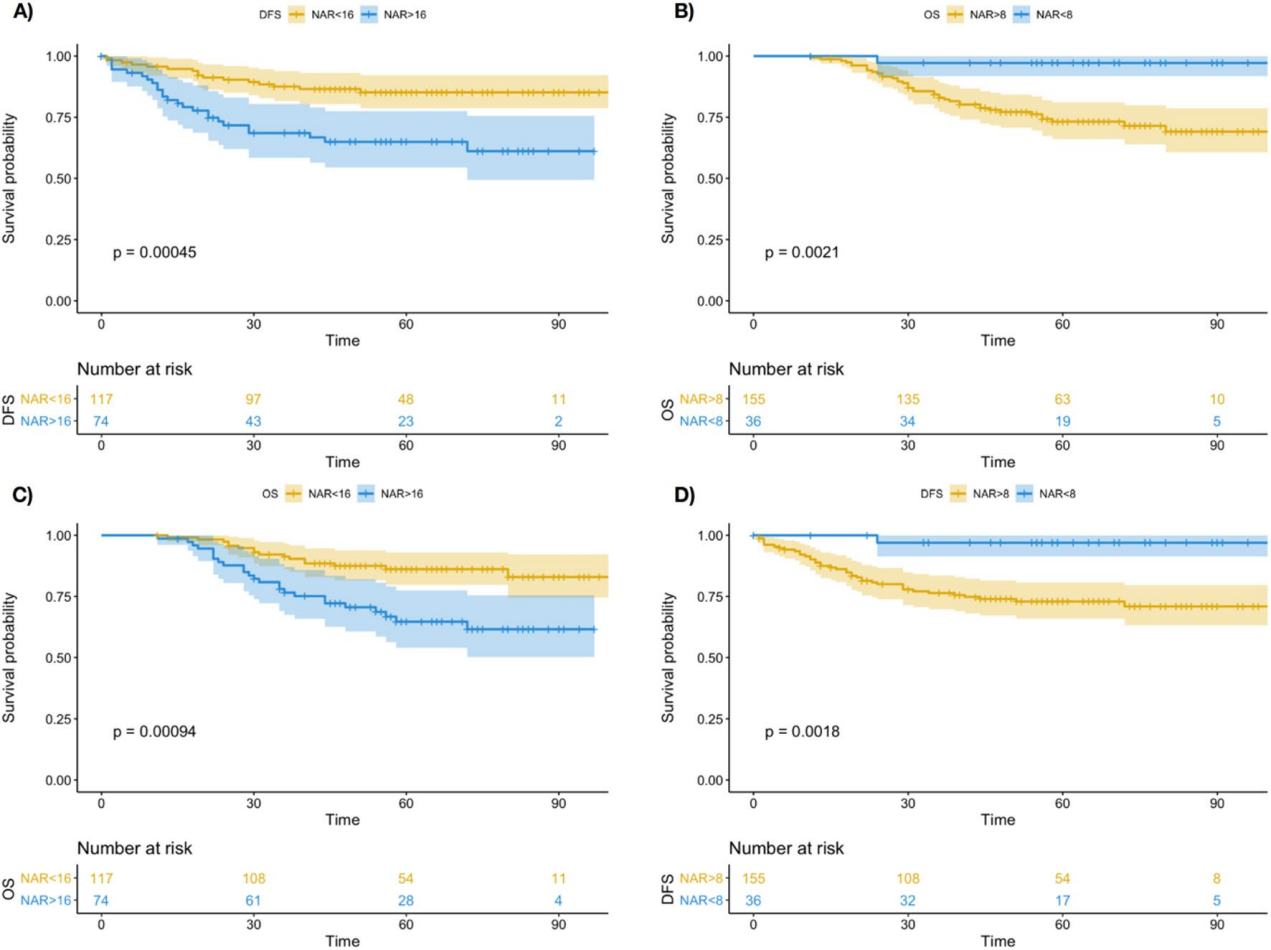
The Kaplan–Meier Survival Curve for (**A**) DFS for NAR > 16 versus NAR < 16, (**B**) OS for NAR > 8 versus NAR < 8, (**C**) OS for NAR < 16 versus NAR > 16 and (**D**) DFS for NAR > 8 versus NAR < 8.

The NAR score ranged from 0 to 65 (IQR, 8–30; median, 15). When binned into categories 0 (0 > 8), 1 (8–16), and 2 (> 16), there were 19% (36/ 132), 42% (81/132), and 39% (74/191) of categories 0, 1, and 2, respectively. For this study, we focused on building predictive radiomics model for the high NAR (> 16) and low NAR (< 8) cohort. Kaplan–Meier survival curves were plotted in Fig. 2 for the DFS and OS of NAR > 16 vs NAR < 16 and NAR > 8 vs NAR < 8. A log-rank test shows statistically significant difference in the OS and DFS of the two cohorts (*P* < 0.01). This shows deriving a clinico-radiomics model for NAR prediction is helpful for determining the high-risk and low risk group during diagnosis. The breakdown of patients of high NAR (> 16) and low NAR (< 8) groups are also summarized in Table 1. The patient survival characteristics are summarized in Table 2.

### Image acquisition, segmentation and radiomics feature extraction

150 patients were scanned with GE CT Scanner while the remaining 41 patients were scanned with Siemens scanner. All the 191 patients have the GTV and CTV segmented. Figure 1 shows the schematics of the analysis pipeline including covariates definitions and modelling methodologies. Radiomics features comprising of first order, textural and shape descriptors are extracted from the manually segmented GTV and CTV. Features that pass the robustness frameworks are input into the survival and NAR models together with the clinical features.

### NAR and survival model building and performance

#### NAR model

**Figure 3:**
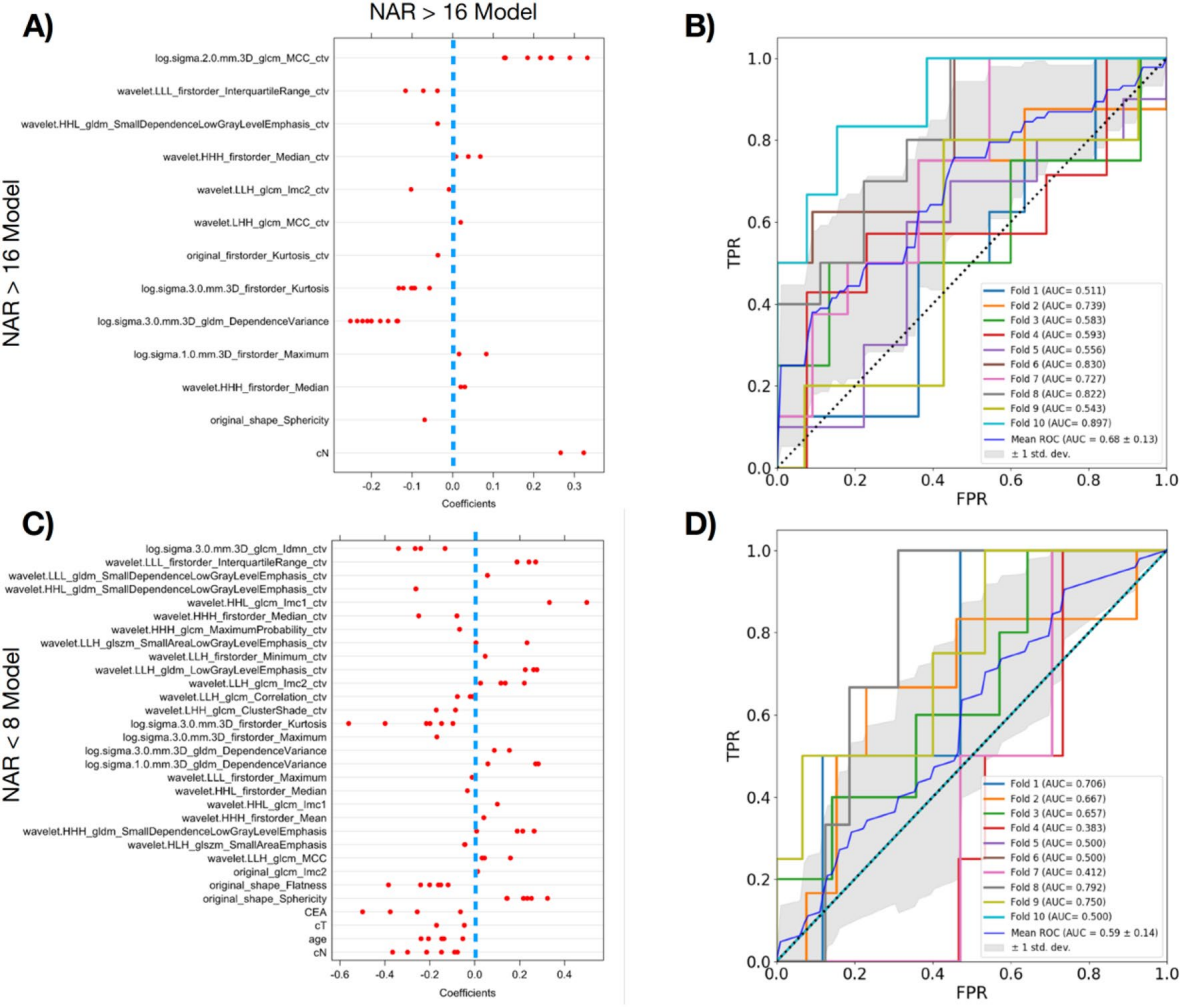
(**A**,**C**) The LASSO coefficients of the radiomics and clinical features in the NAR model for each of the fold in cross validation. Each red dot represents the coefficient of the feature in the model for a particular fold. The blue dotted line corresponds to coefficient 0. (**B**,**D**) This figure shows the 10 ROC curves for each of the 10 folds and final average ROC curve and the associated 95% confidence interval band in gray.

**Figure 4:**
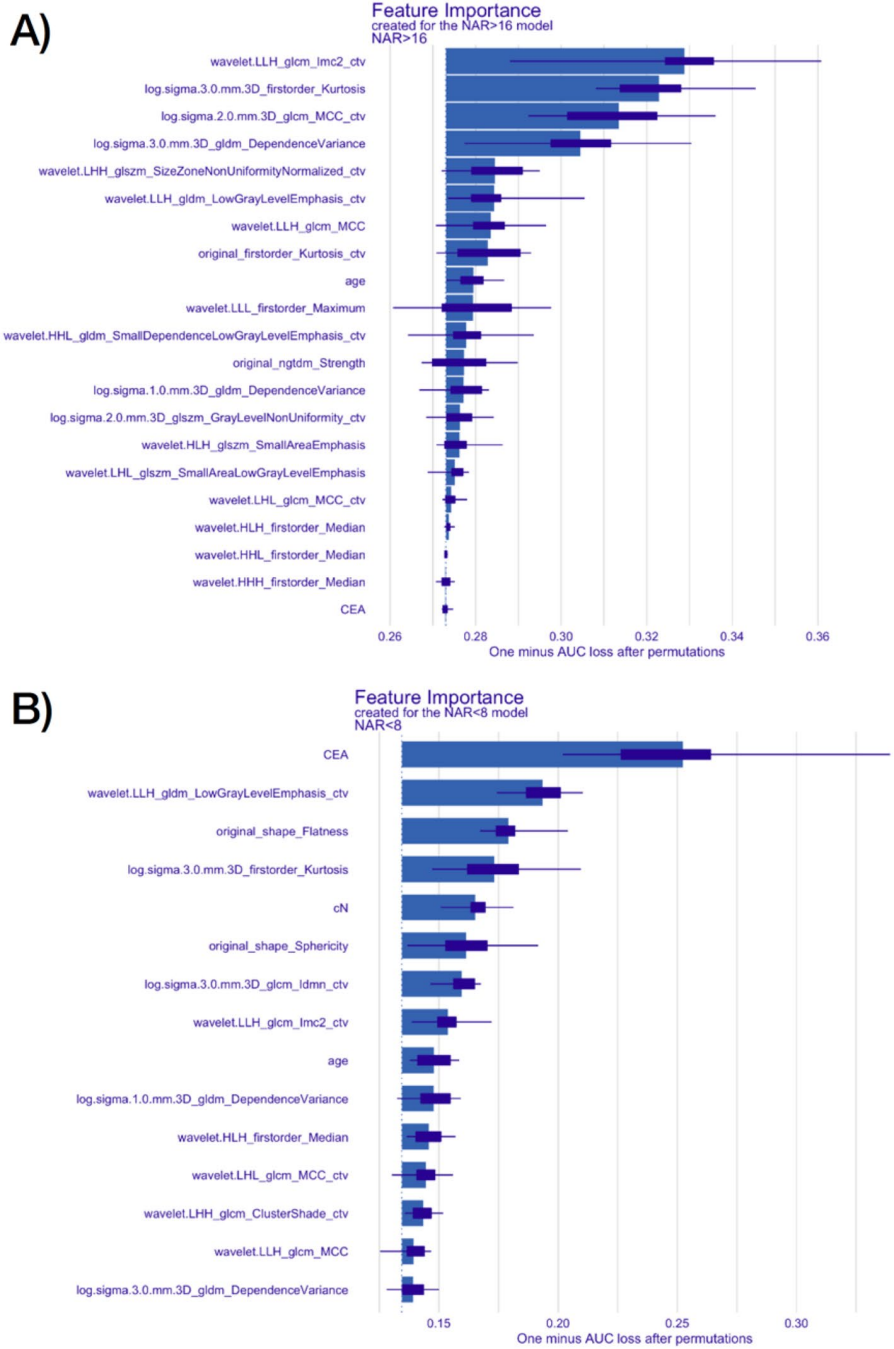
The global feature importance plots using the DALEX package for (**A**) NAR > 16 and (**B**) NAR < 8 prediction model. The radiomics feature with a “ctv” suffix represents features extracted from the CTV segmentation while those without are extracted from the GTV segmentation.

The coefficients of the NAR > 16 model based on LASSO logistic regressions are shown in Fig. 3A. cN is the only clinical feature selected by LASSO algorithm in two of the folds. Two of the radiomics features show strong relation to the outcome and are consistently selected in all the folds—GLCM_MCC_ctv and GLDM_DependenceVariance. The ROCs of all the folds are shown in Fig. 3B. Similarly, the coefficients and ROC curves of the NAR < 8 are shown in Fig. 3C and D respectively. There are more features compared to the NAR > 16 model due to lesser events and greater variation in the selected features in each fold. All the four clinical features (CEA, cT, age and cN) are selected frequently in the model. The feature importance plots for NAR > 16 and NAR < 8 is shown in Fig. 4A and B respectively. The features at the top of the plot are more important in the model and correspond to higher AUC loss when the values are randomized. Radiomics features from the CTV are ranked higher than those extracted from GTV in both models. This shows that CTV radiomics features are more important than the GTV features for the NAR model. The most important clinical features in the combined models as seen from the feature importance plots in Fig. 4, are age and CEA for NAR > 16 and NAR < 8 models respectively. The average AUC of all the folds in the NAR > 16 and NAR < 8 model are 0.68 ± 0.13 and 0.59 ± 0.14 respectively. The probability cutoff for the final NAR < 8 model is 0.3048 and the sensitivity, specificity, PPV and NPV are 0.75, 0.55, 0.28 and 0.91 respectively. The probability cutoff for the final NAR > 16 model is 0.3936 and the sensitivity, specificity, PPV and NPV are 0.68, 0.72, 0.60 and 0.78 respectively. These final models are trained and evaluated on the entire dataset. An additional analysis is also conducted to compare the current approach in NAR modelling with the radiomics score approach (which is used in survival modelling). The result is shown in Fig. S3 where no significant difference is observed between the two approaches.

#### Survival model

**Figure 5:**
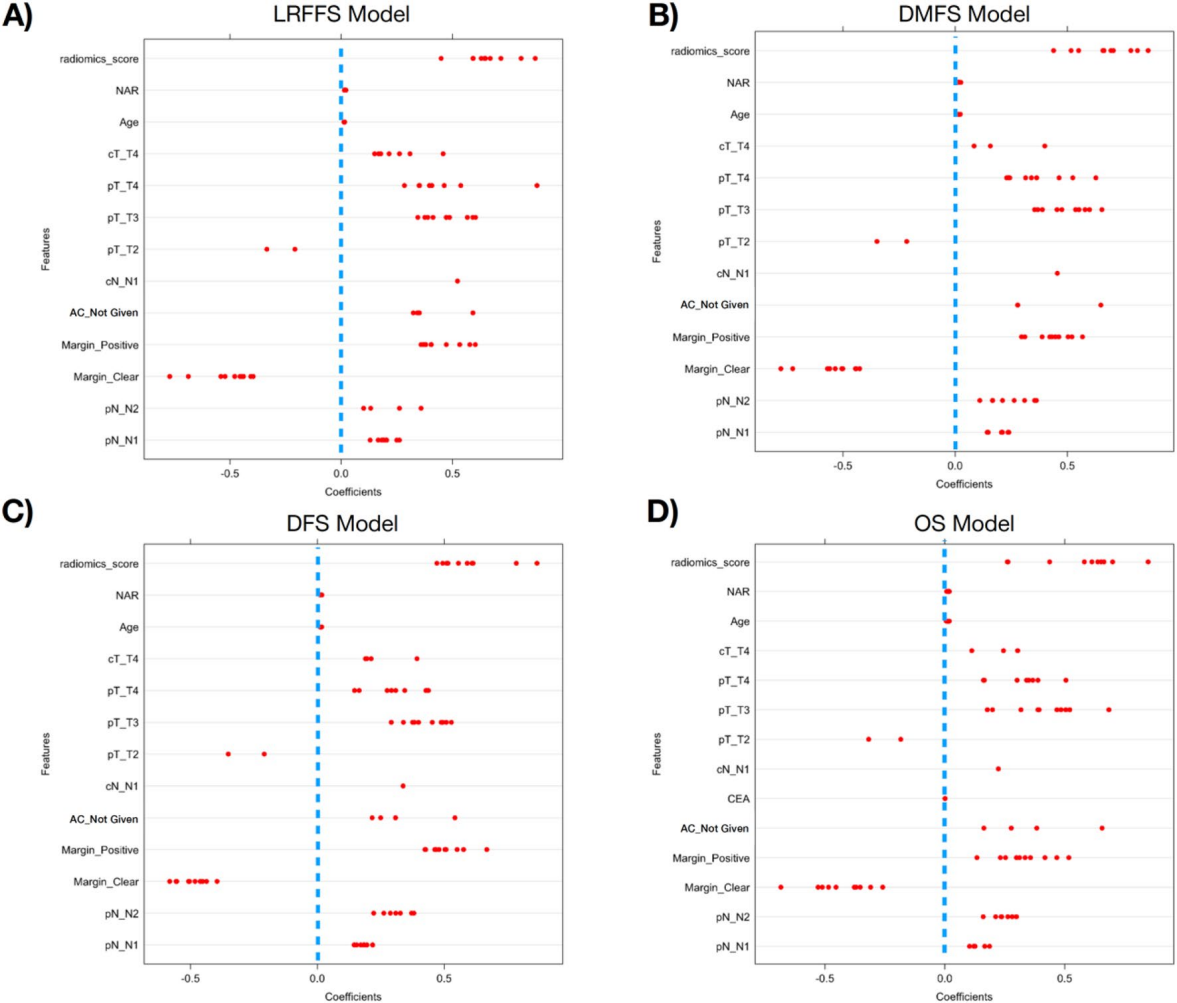
The coefficients of the combined ridge regression models for (**A**) LRFFS, (**B**) DMFS, (**C**) DFS and (**D**) OS for the 10 folds cross validations. Each red dot represents the coefficient of the feature in the model for a particular fold. The blue dotted line corresponds to coefficient 0.

The coefficients of the combined ridge regression models in the survival modelling are shown in Fig. 5. In all the survival modelling, radiomics score has a consistently high coefficient value in all the folds, which corresponds to a high hazard ratio. The plots of the coefficients of radiomics features for this model are shown in Fig. S4. The significant clinical features which are selected consistently in at least 7 of the 10 folds are age, NAR score and margins across all the various survival outcomes.

**Figure 6:**
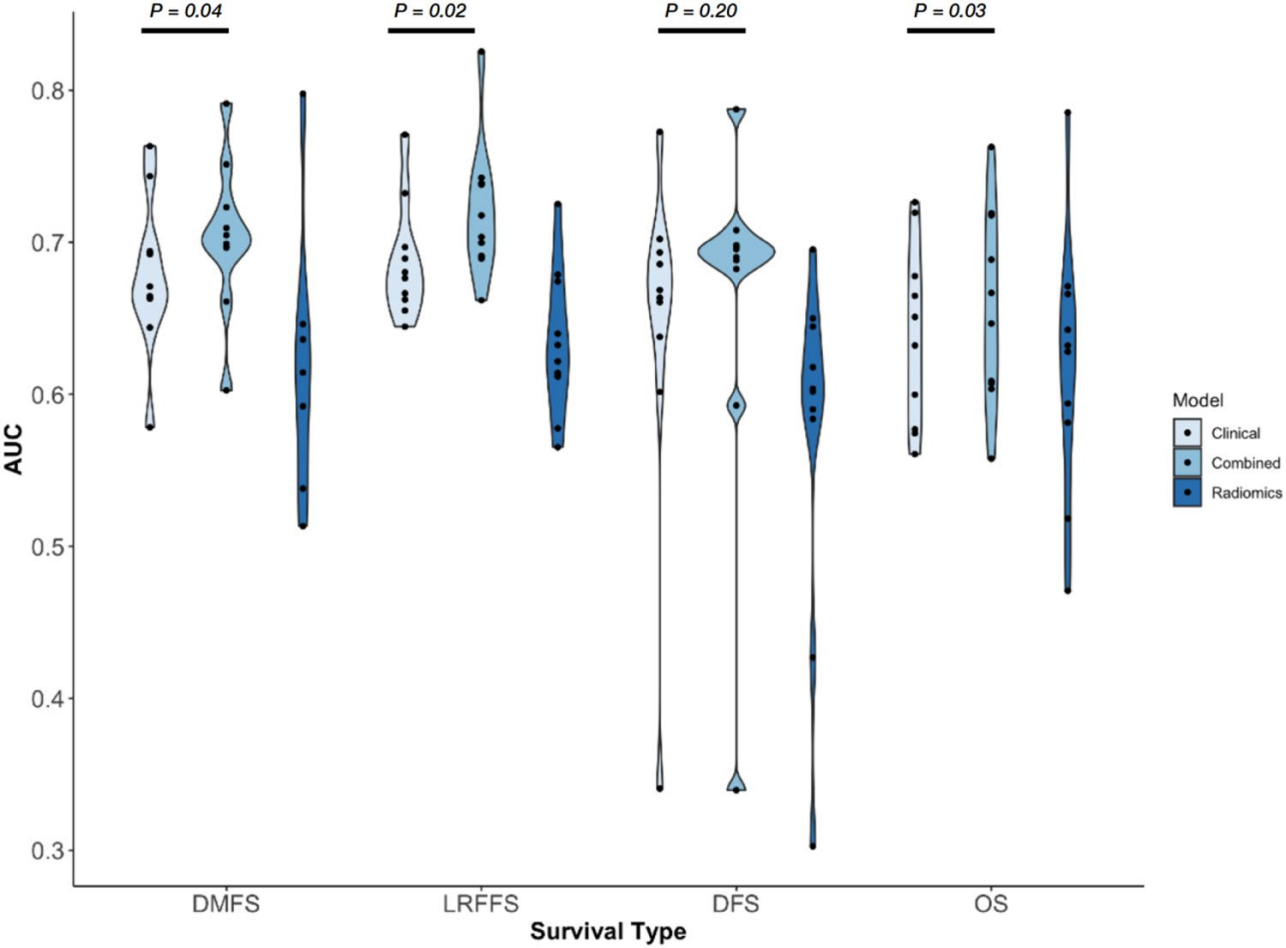
The violin plot of the time-dependent AUCs obtained for radiomics, clinical and combined models for the 4 types of survival. The *P* values are shown on top between the clinical and combined models. DMFS, LRFFS and OS show statistically significant difference between the combined and clinical model.

The time dependent AUCs of the clinical, radiomics and combined models in all the folds are shown in Fig. 6. The AUCs of the clinical, radiomics and combined model of LRFFS are 0.68 ± 0.04, 0.63 ± 0.05 and 0.72 ± 0.04 respectively. The AUCs of the clinical, radiomics and combined model of DMFS are 0.68 ± 0.05, 0.63 ± 0.08 and 0.70 ± 0.05 respectively. The AUCs of the clinical, radiomics and combined model of DFS are 0.64 ± 0.11, 0.57 ± 0.12 and 0.66 ± 0.12 respectively. The AUCs of the clinical, radiomics and combined model of OS are 0.64 ± 0.06, 0.62 ± 0.09 and 0.66 ± 0.06 respectively. The violin plot of the AUCs of the clinical, radiomics and combined models for the four different survivor outcomes is shown in Fig. 6. Pairwise *t-tests* show statistically significant improvement in AUC for LRFFS, DMFS and OS after inclusion of radiomics features on top of clinical features. Even though there is only a modest increase in the mean AUCs of DMFS and OS of 0.02, the paired *t-test* is statistically more power than *unpaired test* and able to detect differences amid large variation between the different folds.

## Discussion

In this study, we investigated the feasibility of predicting NAR score and survival outcomes for LARC patients using deep machine learning and radiomics modelling constructed from radiotherapy planning contrasted CT images. The results indicate that the radiomics features can augment the predictive power of clinical models for OS, DMFS and LRFFS. The model was able to predict these outcomes with moderate accuracy.

The challenge with LARC is that most validated predictive and prognostic models are based on post-operative parameters, limiting the ability for pre-operative treatment decisions. There is emerging data that the response to standard NACRT is heterogenous^4^. With about 20% of patients achieving pCR after standard NACRT, the indications for life changing surgery for this group of patients require justification, especially with data supporting good outcomes when a watch-and-wait strategy was adopted^13,14^. On the other hand, some patients do not respond adequately to standard NACRT. For these patients, an intensified strategy such as that described in the RAPIDO trial or adjuvant chemotherapy may be more appropriate^11^. This is where the radiomics prediction model can be utilized for personalized patient-centred pre-operative treatment decision making.

This study also showed that the radiomics model predicted the NAR score with moderate accuracy. Furthermore, in our study, we correlated the NAR score with survival outcomes and this congruently indicated that the higher the NAR score, the poorer the outcome. Most radiomics studies in LARC predict for pCR which is a dichotomous histopathologic variable achieved in only about 20% of patients post-NACRT^16,22^. In comparison, the NAR score which is derived from more variables, may provide more information. The NAR score is a widely used surrogate in clinical trials^19^. It was developed and widely validated as a short-term endpoint to act as surrogate for DFS and OS in rectal cancer to allow more rapid determination of success or failure of an experimental intervention in LARC^19,41–44^. The NAR score has a greater predictive ability than pCR for OS^19,42^. From the NSABP R-04 randomised phase 3 trial patient dataset, the authors conclude that the 5 year OS for NAR < 8 (low), NAR 8–16 (intermediate) and NAR > 16 (high) were 92%, 89% and 68% respectively^42^. In the German CAO/ARO/AIO-04 randomised phase 3 trial patient dataset, they found that the 3 year DFS was 91.7%, 81.8% and 58.1% for low, intermediate and high NAR score respectively^44^. However, the NAR score can only be calculated after neoadjuvant treatment and resection and is therefore not available to clinicians for making the decision to offer neoadjuvant treatment at the outset. Again, this is where the radiomics model for predicting NAR score can be useful in guiding pretreatment counseling but it may also lend its use in clinical trials.

Our results show that the model has a relatively good discriminatory ability when predicting for high NAR > 16 with an AUC of 0.68 ± 0.11. On the other hand, the NAR < 8 model shows large variation in AUC from 0.383 to 0.792 in Fig. 3D across the 10 folds and can be unstable for clinical use now. Further work involving training with larger datasets or conducting more extensive validations are required before actual clinical application. Despite this shortcoming, we applied the NAR < 8 model to a contemporaneous cohort of patients (N = 31) who declined surgery and found that the majority of the patients (N = 29) were predicted to have NAR > 8 and had poorer overall survival (Fig. S4). Here, the NAR model can be used as an added layer of assessment in deciding on neoadjuvant treatment strategies as discussed. Barring the possibility of contraindications, in this group of patients, surgical intervention may have benefited them. Whilst the OS was not statistically significant between patients with NAR < 8 vs NAR > 8, the limitation here was the small sample size and large proportion of patients with NAR > 8 (n = 29) vs NAR < 8 (n = 2) for it to be meaningful and representative. Testing this model on a larger sample size is required but was beyond the scope of this study.

In this study, we demonstrated that the CT based mesorectal (CTV) imaging features contribute significantly to the accuracy of the final model compared to the intratumoral features (Fig. 4). The distinction between intratumoral and peritumoral radiomics has been studied in different cancers^45–49^. Like Shaish et al., we also derived value in the mesorectal compartment in predicting response and prognosis^23^. Most other radiomics studies in LARC often looked at only the gross tumour whilst the mesorectum which contains important information has often been overlooked. The information contained in the peri-tumoral region may inform on immune response, angiogenesis and invasion beyond the usual radiotherapy or surgical fields which in turn can be analysed to additionally predict for survival outcomes^45–49^. This suggests its inclusion in future rectal based radiomics studies with a consideration for further investigations to clinical regions beyond such as the pelvic side wall. The latter may serve as a predictive tool in guiding the need for pelvic lymph node dissection.

We have undertaken several rigorous approaches to ensure the quality of the study. For example, the whole tumour volume and surrounding mesorectum was analysed individually, instead of working with a single segmentation. A robust procedure was designed to select a subset of features from the original 1130 radiomics features to account for CT scanner variation and inter-rater variation in CTV and GTV contouring. A further feature reduction technique based on retaining uncorrelated features was performed. With the eventual final set of 404 and 254 robust radiomics features for the GTV and CTV respectively, this increased the credibility of the study and reduces overfitting with the model. For the model performance, a nested tenfold cross validation was used. Feature reductions were applied strictly to the training fold to ensure no data leakage. The IBSI guide was used in the construction of the model^30^. The overall radiomics quality score (RQS) for our model was 38.89% (Fig. S6), a higher score than most CT-based radiomics where the range is from 0 to 47% with majority falling below 20%^50,51^.

There are several additional strengths to our study. To our knowledge, this is the first machine learning study using contrasted CT-based radiomics of the rectum and mesorectum for the prediction of NAR score and survival outcomes in LARC. We created two radiomics models—the NAR score model and survival model, and compared the relationship between clinical, radiomics and combined features in model performance. The NAR score was also correlated to survival outcomes. Most other CT-based radiomics studies looked at pCR, some of which could not show the added value of radiomics data in predicting pCR or did not additionally predict for survival outcomes^22,52–56^. The international multicentre MRI-based radiomics study by Shaish et al. is the only other radiomics study in LARC predicting for NAR score^23^. Their model had a similar performance (AUC of 0.66) and the study also evaluated the mesorectal compartment. Nevertheless, the methodology was heterogenous with variable MRI scanner, MRI protocol and neoadjuvant chemotherapy used over the accrued time and between institutions. The authors however felt that the heterogenous image data was a strength of their study as they showed that after controlling for imaging parameters in multivariate analysis, the radiomics features bear most of the predictive strength, driving the outcome-response R2 and improves the generalizability of the model. The results obtained from the study may be too optimistic due to data leakage from performing feature selection in a single fold while evaluating the performance using random train-test split.

Most radiomics studies for predicting treatment response and survival in LARC have been MRI-based^28^. Translation of MRI-based radiomics application in real world is often limited by cost, lack of resources, difficulty with reproducibility and lack of multi-centred validation. Even though all imaging modalities do suffer from this data inconsistency problem due to different models and vendors, this is less of an issue with CT imaging and CT-based radiomics as the voxel value (known as Hounsfield Units) has an actual physical interpretation relating to the X-ray attenuation coefficient. The absolute voxel values thus need to have specific values for specified materials when checked during regular quality assurance process; all CT scanners must conform to this international practice^57^. This ensures certain degree of consistency between CT images acquired across different scanners and provides an advantage for using CT-based radiomics. Furthermore, our model is more readily deployable due to the utilization of routinely performed pre-radiation therapy CT scan. The use of contrasted scans in our study may also provide additional textural features^58^.

There are several limitations to this study. All segmentations were performed by a single radiation oncologist which may introduce bias but were nonetheless performed without knowledge of the pathologic outcome of the patient. To account for intra-rater variation in contouring, we mimicked the contouring by dilating and eroding the contours from the single radiation oncologist. This was described in detail in the Supplementary Method. As this was an exploratory study, a retrospective methodology was used, sample size was small and the study was conducted in a single centre with no external validation cohort. Although we used nested cross-validation which is more rigorous than a single hold-out internal test set, this was not as rigorous as external validation. Future work will involve applying the model to CT data acquired from a different institution to assess the generalizability of the model. Finally, we recognize that different institutions may use different software platforms making it difficult to compare or reproduce results. We recommend standardization of radiomics workflow, use commercially available software and avoid in house applications between institutions.

There are two main recommended ways our model can be used in the real world setting. At the outset, discussions such as more intensified neoadjuvant treatment or the possibility of ‘watch-and-wait’ approach post-neoadjuvant treatment can be better guided using both the NAR and survival model. The NAR model can additionally be used in prospective studies or trials when investigating a new neoadjuvant treatment especially when there is difficulty in recruiting participants. This model can be used to predict for NAR score for the included patients, forming the control arm. The same cohort of patients will undergo the experimental treatment and will derive a final NAR score, forming the experimental arm. Comparisons of the NAR score can then be made between the two groups. Future studies also calls for external validation and collaboration among various institutions to create a large annotated dataset to facilitate the establishment of reliable radiomics models. Further evaluation in randomized clinical trials followed by its implementation within treatment planning systems in radiation oncology to better personalize treatments should be considered.

Radiomics is part of the novel multi-omics approach in understanding and improving the management of cancer. An innovative application of these data includes combining one -omic feature with another to enhance the overall performance of data models that guide therapeutic decisions^59^. For example, radiogenomics is a growing field whereby radiomics data is mined to detect correlations with genomic patterns to provide diagnostic and prognostic imaging biomarkers to guide personalized treatment. In rectal cancer, preliminary studies have shown promising associations between radiomic features and genetic profiles which in turn predict for treatment response and prognosis^59–61^. Whilst multi-omics studies has been conducted in other cancer subsites such as lung cancer^62^, there are no studies looking at the integration of radiomics with other -omics such as proteomics, metabolomics and transcriptomics in rectal cancer. These represent an unmined field with great potentials.

## Conclusions

A radiomics model using pretreatment radiotherapy planning CT images can predict for NAR score and survival outcomes in patients with locally advanced rectal adenocarcinoma undergoing neoadjuvant treatment and total mesorectal excision. Both the tumor and surrounding mesorectal compartments contain important information for predicting response. The resulting information can aid clinicians in risk stratifying patients which may improve patient selection to the different treatment options such as varying the neoadjuvant approach, adding or intensifying adjuvant therapy, altering the surgical approach and determining surveillance interval. Further prospective studies are required to validate this model and evaluate its implementation within treatment planning systems.

## Supplementary Information

Supplementary Information.
