# Supplementary material for: Predicting outcomes for locally advanced rectal cancer treated with neoadjuvant chemoradiation with CT-based radiomics
Source: Sci Rep. 2022 Apr 13;12:6167. doi: 10.1038/s41598-022-10175-2 (PMC9008122; doi:10.1038/s41598-022-10175-2)

**Supplementary Materials**

Table of Contents

[Supplementary Methods 1](#_Toc93835406)

[Statistical Toolboxes Used in this work 1](#_Toc93835407)

[Robust Radiomics Features Selection 1](#_Toc93835408)

[Applying NAR Model on Patients Without Surgery 2](#_Toc93835409)

[Supplementary Figures 3](#_Toc93835410)

[Figure S1: Phantom set-up for Radiomics Feature Robustness Study 3](#_Toc93835411)

[Figure S2: Voxel HU distributions for different Scanners and Settings 4](#_Toc93835412)

[Figure S3: Comparison of radiomics score against full radiomics features approach for NAR modelling 5](#_Toc93835413)

[Figure S4: Coefficients of Radiomics features for Survival Model 6](#_Toc93835414)

[Figure S5: Stratified Kaplan Meier curves of the patient cohort who did not undergo surgery. 7](#_Toc93835415)

[Figure S6: The answers to the questionnaire used for calculating the Radiomics Quality Score (RQS) 8](#_Toc93835416)

# Supplementary Methods

## Statistical Toolboxes Used in this work

The Kaplan-Meier survival curves were calculated and plotted using *survminer_0.4.8.* The feature reduction method by removing correlated features were performed using *findCorrelation* in *caret_6.0-84*. The relief algorithm was implemented using *attrEval* in *CORElearn_1.53.1*. LASSO logistic regression and regularized Cox regression were performed using *glmnet_2.0-18*. Global feature importance was calculated with *DALEX_2.0.* Time dependent AUCs were calculated using *survAUC_1.0-*5.

## Robust Radiomics Features Selection

The robust radiomics features were determined by assessing the effect of perturbations introduced to the CT image on the feature value. Feature value that was relatively unchanged upon perturbation was considered robust and the Intra-class correlation (ICC) metric was used for this purpose. The perturbations considered were 1) contour perturbation and 2) noise addition. Contour perturbation include a 1 mm and 2 mm dilatation and erosion operation on the segmentations to mimic the effect of inter-rater variations. The noise addition was to simulate the effect of scanner variation. The amount of noise to be added was determined from our phantom study.

A CIRS phantom was scanned using both the Siemen and GE CT scanner in our department, and several Region-of-interest (ROI) was identified in the phantom. This is illustrated in Figure S2. Histograms or distributions of the HU values within the ROI were consolidated for different scanners and different scanning parameters. Assuming the voxels are spatially independent, the difference between the distribution of the GE and Siemen scanner in the ROI will indicate the “noise distribution” to be added to account for the scanner variation. The difference can be calculated by a convolution operation between the two distributions. The result is shown in Figure S3. It can be seen that the “noise distribution” is fairly similar for different kVp and mAs settings. This noise distribution has a mean of 20 HU and standard deviation of 20 HU. Hence, these mean and standard deviation parameters were adopted for the parametric noise distribution.

Lastly, different parametric noise distribution such as Gaussian, Poisson and Uniform noises were added to the CT data to simulate the effect of scanner variation. Due to the random nature of the noise distribution, radiomics feature was extracted five times for each type of noise added to the CT image. This results in 19 sets of radiomics features per patient (15 from noise addition to the CT image and 4 from morphological operation on the segmentations). ICC was then used to assess the stability of the feature value across the 19 sets of radiomics features.

## Applying NAR Model on Patients Without Surgery

The final NAR<8 model was trained on the entire 191 patients’ cohort and tested on an independent group patients who did not undergo surgery. The optimal cutoff for the model was chosen based on the Youden’s index and corresponds to 0.40. Of the 31 patients without surgery, 29 of them were predicted to be NAR>8 while the remaining 2 were predicted to have NAR<8.

# Supplementary Figures

## Figure S1: Phantom set-up for Radiomics Feature Robustness Study


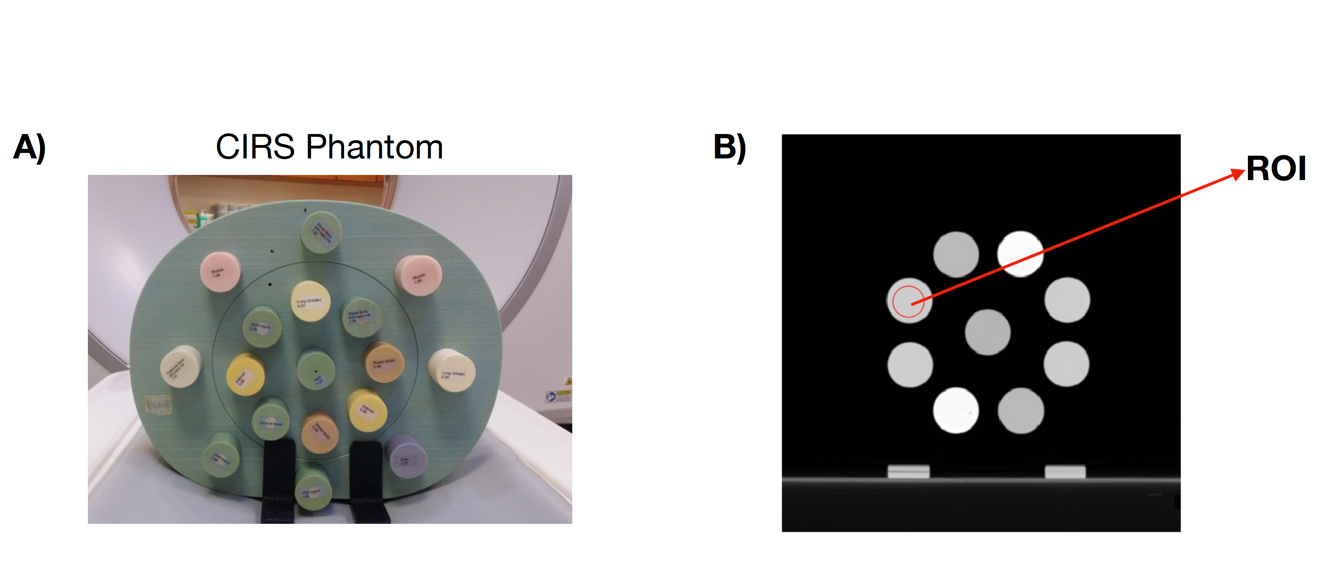


##

## Figure S2: Voxel HU distributions for different Scanners and Settings


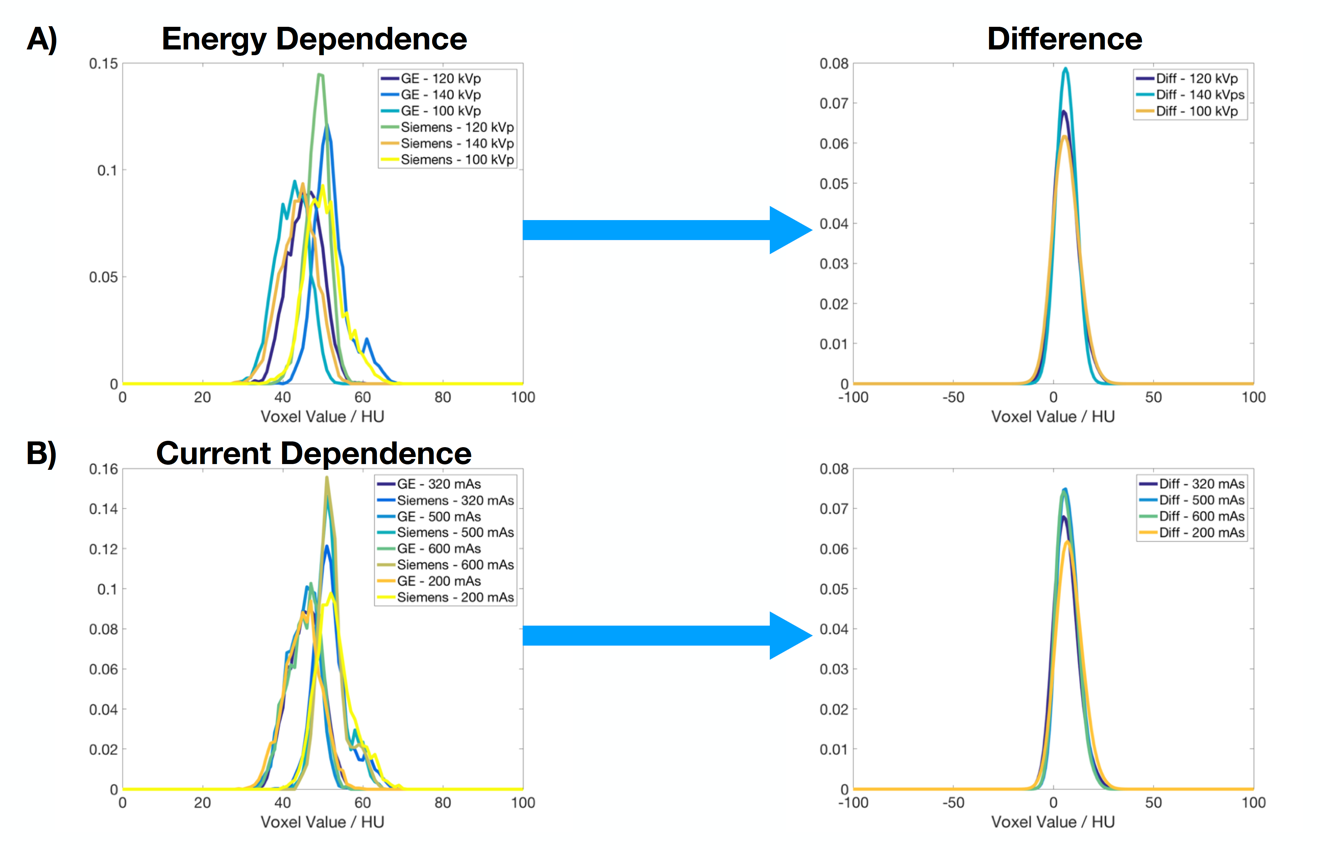


A) Distribution of the voxel value within ROI for different CT scanner under different kVp. The difference between the distribution of the GE and Siemens scanner under the same kVp are calculated by performing convolution between the two distributions from different scanners. B) Distribution of the voxel value within ROI for different CT scanner under different mAs. The difference between the distributions are calculated using convolution.

## Figure S3: Comparison of radiomics score against full radiomics features approach for NAR modelling

##
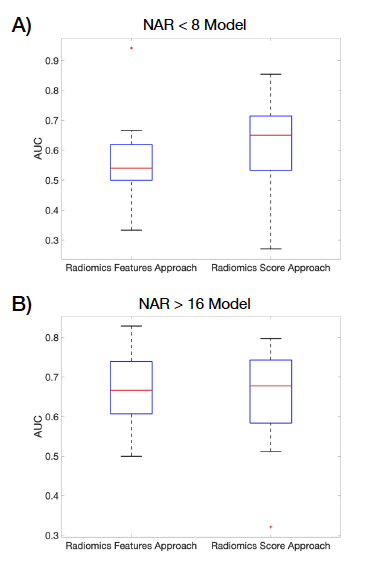


## Figure A and B show the comparison of the radiomics score versus radiomics features approach in NAR<8 and NAR>16 models respectively. Both approaches are used to construct the NAR models and the AUCs across the 10 folds are shown in the box plots. Paired t-tests were carried out but no statistical significance between the approaches were observed. The AUCs across the folds in the radiomics feature approach differ slightly from Figure 3 due to the different fold splits arising from a different random seed. However, this does not affect the result in this comparison as the same folds are subjected to both approaches simultaneously.

##

## Figure S4: Coefficients of Radiomics features for Survival Model


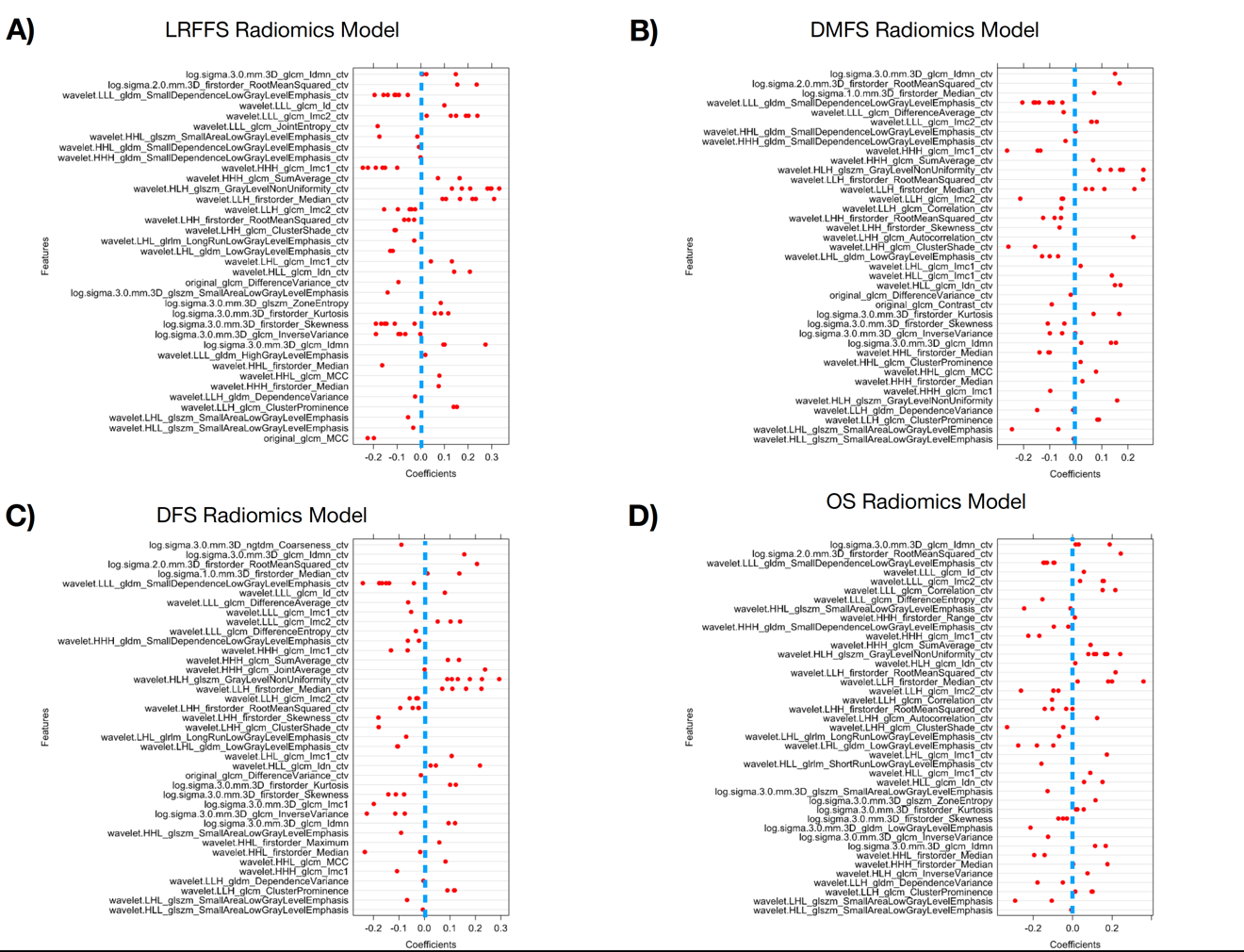


## Figure S5: Stratified Kaplan Meier curves of the patient cohort who did not undergo surgery.


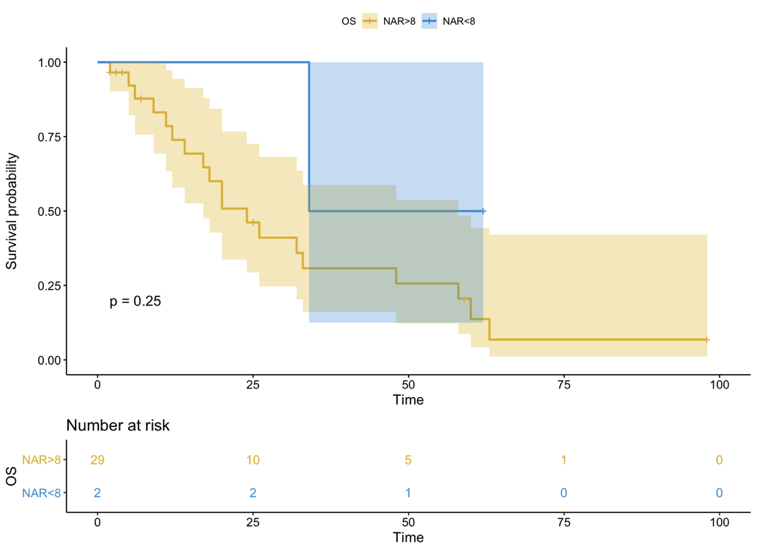


## Figure S6: The answers to the questionnaire used for calculating the Radiomics Quality Score (RQS)

**
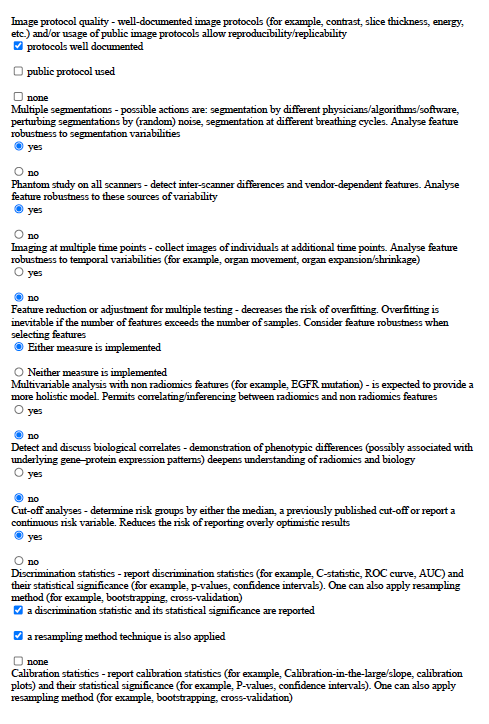
**


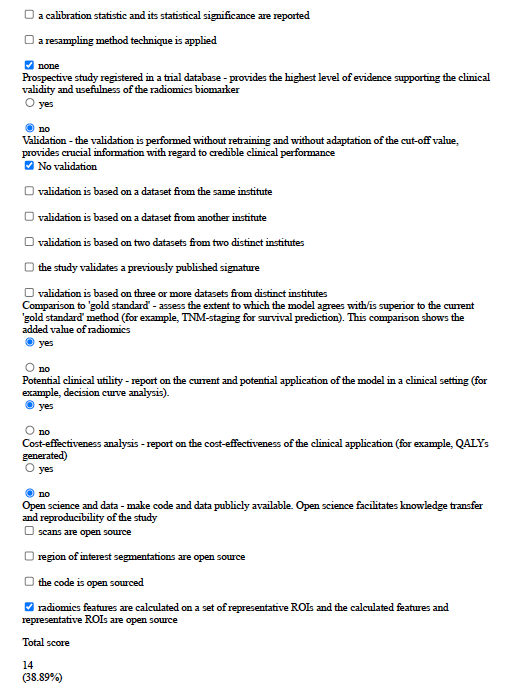

Supplement: Supplementary file 1 — Supplementary Information. [file 41598_2022_10175_MOESM1_ESM.docx]
